# Evaluation of Agronomic Traits, Total Phenolic Content, and Antioxidant Properties of Sesame Seeds of Different Colors and Origin

**DOI:** 10.3390/foods13182932

**Published:** 2024-09-16

**Authors:** Collins Yeboah Osei, Sookyeong Lee, Gi-An Lee, Sae Hyun Lee, Eunae Yoo, Jae-Eun Lee, Eun-Gyeong Kim, Tae-Jin Yang

**Affiliations:** 1 National Agrobiodiversity Center, National Institute of Agricultural Sciences, Rural Development Administration, Jeonju 54874, Republic of Korea; coyeboah@wacci.ug.edu.gh (C.Y.O.); xsanta7@korea.kr (S.L.); gkntl1@korea.kr (G.-A.L.); eung77@korea.kr (E.Y.); jnlee88@korea.kr (J.-E.L.); keg950@korea.kr (E.-G.K.); 2 Council for Scientific and Industrial Research, Plant Genetic Resources Research Institute, Bunso P.O. Box 7, Ghana; 3 Department of Agriculture, Forestry and Bioresources, Plant Genomics and Breeding Institute, Research Institute of Agriculture and Life Science, College of Agriculture and Life Sciences, Seoul National University, Seoul 08826, Republic of Korea; whyskyisgray@snu.ac.kr

**Keywords:** antioxidant properties, agronomic traits, sesame, seed color, origin

## Abstract

Rising health concerns regarding chronic diseases call for exploring natural sources of antioxidants and factors that influence their activity. This study evaluated the diversity of 112 sesame germplasms from Africa and Asia based on ten agronomic traits (seven quantitative and three qualitative), two antioxidant activities (ABTS and DPPH radical scavenging activities), and the content of one metabolite (TPC). TPC, DPPH, and ABTS were in the ranges of 4.98–87.88 µg GAE/mg DE, 3.97–46.23 µg AAE/mg DE, and 3.42–176.01 µg TE/mg DE, respectively. Statistical analyses revealed significant variations in agronomic traits, TPC, and antioxidant activities among the sesame germplasms (*p* < 0.05). Furthermore, the individual and interaction effects of seed color and the continent of origin on the levels of the quantitative traits, TPC, ABTS, and DPPH were analyzed, and the correlation among the traits was further evaluated. Diversity in TPC, ABTS, and DPPH was significantly associated with seed color and most of the quantitative agronomic traits (*p* < 0.05) but not with continent of origin. Principal component analysis revealed TPC, ABTS, DPPH, and five quantitative traits as the most discriminant traits. In general, six sesame accessions with high TPC and antioxidant activities (IT194356, IT170094, IT29971, IT185998, IT104246, and IT169623) as well as important agronomic traits were identified and, hence, could be used for developing improved sesame varieties.

## 1. Introduction

With the heightened awareness of and concerns about chronic health issues including cancer, diabetes, cardiovascular diseases, and chronic inflammation, food preferences and consumption patterns now span beyond just dietary composition. Nowadays, consumers show keen concern about and interest in the health benefits of the food they consume, resulting in a high demand for functional or nutraceutical foods [1,2,3]. This class of foods not only meets the basic energy and nutritional requirements of consumers but also contains bioactive compounds that are useful in enhancing the body’s response to these metabolic and chronic diseases [4,5,6]. For years, several research findings have revealed that cell damage and DNA damage, which result in the development of chronic diseases, are associated with the overproduction of free radicals in contrast with cellular antioxidants in the human body, a phenomenon termed oxidative stress [7,8,9,10]. Endogenous antioxidants produced by the body to neutralize free radicals are sometimes inadequate, creating this imbalance and the ensuing negative effects [9,10]. The scientific response to oxidative stress-related diseases has included directing the focus of research objectives to exploring the biochemical composition of various crop species for natural sources of antioxidants, leading to the discovery of functional foods [11,12,13].

Sesame (*Sesamum indicum* L. 2n = 26) is an erected herbaceous annual crop belonging to the *Pedaliaceae* family [14]. Sesame features as a functional food crop because aside from its nutrient-dense nature, it contains important bioactive compounds including phenols, flavonoids, lignans, melatonin, and tocopherols known to exhibit antioxidant properties [15,16]. These bioactive compounds are associated with therapeutic effects against several chronic diseases [17]. Sesamin and sesamol, the main antioxidant lignans in sesame seeds, have demonstrated efficacy in managing diabetes, hypertension, atherosclerosis, and thrombosis [14,15,18]. Wei et al. [14] highlighted the effectiveness of sesame phytosterols, fatty acids, and lignans in lowering blood pressure. Additionally, sesamin has shown inhibitory properties against cancer cell growth and proliferation, particularly in breast and prostate cancer cells [15]. Sesame seeds are rich in tocopherols, which possess anticancer properties due to their free radical scavenging activities [15,18]. Among oilseed crops, sesame is exceptionally recognized for its high-quality oil (40–60%) and numerous nutritional and health benefits [15,16,19]. In the quest to compile a list of crops classified as Future Smart Foods (FSF) in Myanmar, sesame was duly recognized by Li and Sidiqque [20]. The recognition was based on the nutritional benefits, agricultural relevance, and economic importance of sesame, indicating its relevance in achieving food and nutritional security. Sesame is cultivated mainly in the developing tropical and subtropical areas of Asia and Africa, where wider genetic diversity in the crop also exists [19,21]. The two continents account for 50.3% and 45.4% of global sesame production, respectively [21,22]. Currently, Sudan is the leading producer of sesame with more than 1 million tonnes of production. India and Myanmar follow with 788,740 and 760,925 tonnes of production, respectively [22].

Large collections of sesame germplasms are being conserved in national and international gene banks worldwide [23]. However, more comprehensive studies on the diversity present among sesame genetic materials are still scarce. Many of the previous research studies mainly focused on exploring the diversity in their agro-morphological traits, whereas the diversity in their metabolite composition and antioxidant activities remains underexplored [24,25,26,27,28]. The available literature in this regard also used either a small number of germplasms or relied on samples collected from markets or sesame byproducts [29,30,31,32,33]. On the other hand, a pattern of interrelationship among morphological, biochemical, and physiological plant traits and environmental conditions has been reported [30,34,35,36,37]. However, there is not much information on the association of agronomic and environmental factors with the metabolite and antioxidant activities of sesame. Exploring the available diversity in metabolite composition and antioxidant activities among sesame germplasms could serve as an invaluable resource for developing sesame varieties with desirable qualities to be utilized in nutraceutical foods [38]. Accordingly, this study aimed to evaluate the diversity in 112 sesame germplasms originating from Asia and Africa using agronomic traits, total polyphenol content (TPC), and antioxidant activities (ABTS and DPPH radical scavenging activities). Moreover, the main and interaction effects of seed color and origin (continent) variations on the analyzed parameters were investigated to estimate the association between agronomic traits and biochemical parameters. The results of this study could be valuable for the selection and identification of superior sesame accessions to be used in breeding programs and for enhancing their antioxidant potential.

## 2. Materials and Methods

### 2.1. Plant Materials Collection and Cultivation

A total of 112 sesame germplasms conserved at the National Agrobiodiversity Center, Rural Development Administration (RDA, Jeonju, Republic of Korea), were used for this study. The field experiment was conducted in the research field of the National Agrobiodiversity Center, RDA (35°49′18″ N, 127°08′56″ E). Sesame seeds were planted at a distance of 45 cm on single-row ridges covered with 0.02 mm thick black plastic sheets during the spring season on 13 May 2021. All necessary agronomic practices including pest control and irrigation were observed during the growing period. Capsules were harvested at maturity for seed extraction and dried for 3 days at 45 °C in a VS-1202D drying oven (Vision Scientific, Bucheon, Republic of Korea). Dried seeds weighing 10.3–10.5 g were crushed into powder and stored at −20 °C in test tubes pending further analysis. The germplasms were classified based on their continent of origin. Accordingly, 71 accessions were from Asia while the remaining 41 accessions were from Africa. The germplasms were also classified as black (n = 25), brown (n = 19), light brown (n = 21), olive (n = 6), or white (n = 41) based on their seed color. Detailed information regarding the classification of the sesame germplasms is provided in Table S1.

### 2.2. Agronomic Traits Characterization

The sesame germplasms were phenotypically characterized based on 3 qualitative and 7 quantitative agronomic traits [39]. The quantitative traits included the following: days to flowering (DTF: counted from sowing date to date on which ≥ 50% of the plants initiated flowering), days to maturity (DTM: counted from sowing date to date on which 75% of plants reached physiological maturity), height to the first capsule (HTFC: average length in cm measured from the soil surface to the position of the first capsule-bearing node in 5 plants), capsule zone length (CZL: average length in cm measured from the first capsule-bearing node to the last capsule-bearing node in 5 plants), capsule length (CL: average length in cm of 5 capsules selected randomly from the middle of the main stem), capsule width (CW: average width in cm of 5 capsules selected randomly from the middle of the main stem), and thousand seed weight (TSW: weight in g of 1000 seeds randomly counted from bulk harvest). The qualitative traits were as follows: number of capsules per axis (NCPA: 1: one, 2: three, 3: mixture of one and three capsules), number of locules per capsule (NLPC: 1: four, 2: six, 3: eight, 4: mixture of eight and six, 5: mixture of four and six, 6: mixture of four and eight, 7: mixture of four, six, and eight locules), and seed color (SC: 1: white, 2: black, 3: light brown, 4: brown, 5: olive) [21].

### 2.3. Chemicals and Reagents

Analytical-grade chemicals and reagents were used in this study. All of them, including Folin–Ciocalteu phenol reagent, sodium carbonate (10% Na_2_CO_3_), sodium hydroxide (NaOH), 75% ethanol, gallic acid, ascorbic acid, hexane, 2,2′-azino-bis(3-ethylbenzothiazoline-6-sulfonic acid) (ABTS), 1,1-diphenyl-2-picrylhydrazyl (DPPH), boron triflouride (BF), potassium persulfate (K_2_S_2_O_8_), and Trolox (Hydroxy-2,5,6,8-tetramethyl), were procured from Sigma Aldrich (St. Louis, MO, USA).

### 2.4. Determination of Total Phenolic Content and Antioxidant Activities

#### 2.4.1. Extract Preparation from Sesame Seeds

The procedure for extraction followed a previously reported method with slight modifications [40]. Briefly, 7 g of the powdered sesame seed sample was mixed with 75% ethanol (20 mL) in an extraction tube and prepared for extraction using an accelerated solvent extractor (ASE-350; Dionex, Sunnyvale, CA, USA). The solution was transferred into another tube and concentrated for 10 h at 40 °C using a vacuum concentrator (HT-6; Genevac, Ipswich, UK). For TPC and antioxidant activity analyses, the concentrated extracts were further diluted with 75% ethanol to achieve final concentrations of 2 mg/mL and 0.2 mg/mL, respectively. Individual samples were extracted in triplicate.

#### 2.4.2. Determination of Total Polyphenol Content (TPC)

The total polyphenol content was determined according to previously described methodologies [40,41]. Initially, 100 µL of the sesame seed extract was reacted with 50 µL Folin–Ciocalteu reagent and 750 µL of water. The solution was vortexed and incubated in the dark for 3 min at room temperature. Afterward, the solution was treated with 1 mL of 10% Na_2_CO_3_, vortexed, and again incubated in the dark for 30 min. After the reaction period, the absorbance was measured at 750 nm using an Eon Microplate Spectrophotometer (Bio-Tek, Winooski, VT, USA). TPC was computed as gallic acid equivalence (µg) per mg of dried seed extract (µg GAE/mg DE) using gallic acid as standard.

#### 2.4.3. 2,2′-Azino-Bis(3-Ethylbenzothiazoline-6-Sulfonic Acid) Diammonium Radical Cation (ABTS+) Scavenging Activity

The ABTS scavenging activity was investigated following previously described protocols by preparing a reagent of 7 mM ABTS and 2.45 mM K_2_S_2_O_8_ [40,42]. The reagent was wrapped with foil and refrigerated for 12–16 h. It was further diluted with water to obtain an absorbance of 0.70 ± 0.02 at 734 nm. Afterwards, 10 µL of the sesame seed extract was mixed with 190 µL of ABTS solution and incubated for 6 min in the dark. Absorbance was measured at 734 nm using an Eon Microplate Spectrophotometer (Bio-Tek, Winooski, VT, USA). Trolox was used as the standard and ABTS radical scavenging activity was expressed as µg of Trolox equivalent antioxidant capacity per mg of dried seed extract (µg TE/mg DE).

#### 2.4.4. 1,1-Diphenyl-2-Picrylhydrazyl (DPPH) Radical Scavenging Activity

DPPH free radical scavenging capacity of the sesame seed extract was evaluated according to previously reported methodologies [40,42]. Briefly, a mixture of 100 µL sesame seed extract and 100 µL of DPPH (150 µM) was homogenized. The mixture was incubated in the dark for 30 min at room temperature. The absorbance of the sample was measured at 517 nm using an Eon Microplate Spectrophotometer (Bio-Tek, Winooski, VT, USA). The DPPH radical scavenging capacity was measured as µg of ascorbic acid equivalent antioxidant capacity per mg of dried seed extract (µg AAE/mg DE).

### 2.5. Statistical Analysis

All experiments were replicated three times, and results were expressed as mean ± standard deviation (SD). The data obtained were subjected to statistical analyses including analysis of variance (ANOVA), principal component analysis, cluster analysis, correlation, and path analysis. LSD was computed to statistically determine significant differences between parameters at a level of *p* < 0.05. All analyses were computed using R-software (version 4.2.2).

## 3. Results and Discussion

### 3.1. Diversity in Sesame Germplasms Based on Agronomic Traits and Biochemical Parameters

**Figure 1:**
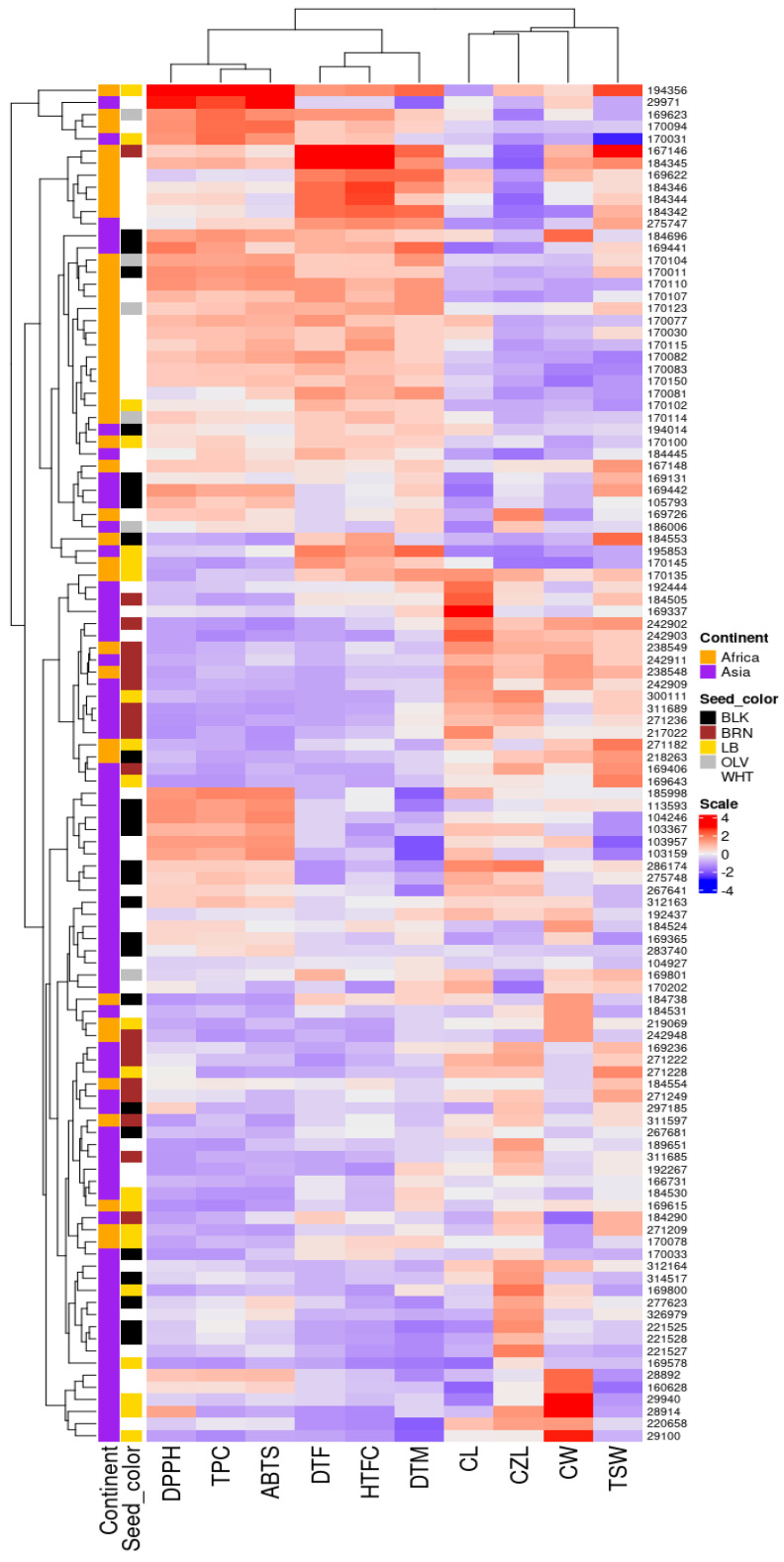
Heatmap of two-way hierarchical cluster analysis of sesame accessions based on continent, seed color, TPC, antioxidant activities, and quantitative agronomic traits. BLK: black, BRN: brown, LB: light brown, OLV: olive, WHT: white.

Table 1 summarizes the relative frequencies of the three qualitative agronomic traits evaluated in this study. Figure 1 demonstrated the extent of diversity in the accessions according to continent of origin, seed color, quantitative agronomic traits, TPC, and antioxidant activities. NCPA varied between one and a mixture of one and three, with 72 and 40 accessions, respectively. A total of 33 accessions, out of those with a mixture of one and three NCPA, were from Asia. NLPC was categorized into five distinct groups, with a greater proportion of the accessions (n = 100, %*f* = 89.3) characterized by four locules per capsule. The other categories of NLPC were less represented in the population. Previous studies observed similar variations [28,43]. Diversity in NCPA was wider in Asian accessions compared to African accessions. NCPA and NLPC are important qualitative traits that determine seed yield per plant in sesame. Hence, sesame genotypes with more NCPA and NLPC are more likely to produce higher seed yields compared to those with few NCPA and NLPC [43,44]. Seed color showed wider diversity, with five distinct colors (Table 1 and Figure 1). Similar diversities in sesame seed color have been reported [26,45,46]. The predominant seed color in this study was white, accounting for 41 accessions, followed by black with 25 accessions. Olive-colored seeds were the least represented with only six accessions (Table 1). In agreement with this study, Gedifew et al. [28] also observed more white seeds (71.87%) among sesame accessions. The two-way hierarchical cluster analysis in Figure 1 demonstrated diversity in accessions based on continent and seed color according to the different clusters. Nevertheless, the clusters contained representative accessions from both continents with all the different seed colors. This could be a result of the global introduction and exchange of sesame genetic resources [47] and its export for commercial utilization [48]. The findings imply that seed color and continent of origin did not inherently introduce much diversity into the sesame germplasms, as previously reported [25,26,49].

Concerning the quantitative agronomic traits, the results revealed a wider variation in most of the traits (Table 2). With means of about 58 and 118 days, DTF and DTM were in the ranges of 44–97 and 90–147 days, showing CVs of 19.4 and 11.4%, respectively. The HTFC ranged between 12.2 and 215.0 cm with a mean of 78.5 cm, showing the highest CV (57.5%). The means and CVs for DTF and HTFC (78.5 cm) were higher than the values previously reported [50]. Likewise, CZL, CL, and CW were in the ranges of 25.0–151.4, 2.16–4.10, and 0.5–1.2 cm with means of 88.7, 2.8, and 0.8 cm, respectively. By comparison, CZL had a higher CV (36.7%), followed by CL (12.2%) and CW (7.0%). TSW ranged from lighter (1.8 g) to heavier (4.4 g), with a mean weight of 3.0 g. The length and width of capsules are an indication of their seed yield since longer and broader capsules will naturally contain more seeds, comparatively. In comparison with a previous report on sesame agronomic traits [46], accessions used in this study exhibited higher means of HTFC (78.5 cm), CZL (88.7 cm), and TSW (3.0 g). Moreover, the means and CVs recorded for all the quantitative traits in this study were higher than those reported by Gedifew et al. [28] except for HTFC, CL, and CW (Table 2). The high CV and the range of colors both indicate the wider contribution of quantitative agronomic traits to diversity in the sesame germplasms [23]. In sesame production, quantitative agronomic traits such as TSW, CZL, CL, and HTFC have been found to be directly associated with yield [46,51,52]. A panel of sesame germplasms with inherent diversity in these traits will therefore serve as a valuable source of breeding materials for improving yield [27].

Information on the diversity in antioxidant activities and TPC of sesame is a prerequisite for selecting and breeding sesame varieties with high TPC and antioxidant activities [38]. Although TPC and antioxidant activities of sesame have been reported, several of these reports utilized byproducts, seeds from the market, or fewer accessions of sesame, which did not comprehensively reveal the range of genetic diversity in sesame antioxidant properties and TPC [29,30,31,32,33]. In this study, the antioxidant activities (ABTS and DPPH free radical scavenging activities) and TPC of 112 sesame germplasms originating from Africa and Asia were analyzed. Table 2 presents the summary statistics of the diversity in these traits. Table S1 and Figure 1 give details about individual accessions that recorded higher and lower TPC, ABTS, and DPPH activities within the population. Levels of TPC, ABTS, and DPPH ranged from 4.9 to 87.9 µg GAE/mg DE, 3.4 to 176.0 µg TE/mg DE, and 3.9 to 46.2 µg AAE/mg, with means of 27.9 µg GAE/mg DE, 52.6 µg TE/mg DE, and 14.1 µg AAE/mg, respectively. The CVs recorded for TPC (58.9%), ABTS (66.5%), and DPPH (56.8%) demonstrated a wider diversity in the evaluated sesame germplasms in this study than in previous reports [38]. The germplasms in this study therefore could provide a range of options as sources of nutraceutical or functional foods and breeding materials for enhancing sesame antioxidant potentials.

### 3.2. Effects of Origin and Seed Color on Quantitative Agronomic Traits

The diversity in quantitative agronomic traits of plants can be attributed to many factors. In this study, the individual and interaction effects of continent of origin and seed color on the diversity in quantitative agronomic traits of 112 sesame germplasms were analyzed. The ANOVA results revealed a significant individual effect of continent on DTF, DTM, HTFC, and CZL, while seed color had significant effect on DTF, CZL, CL, and TSW (*p* < 0.05). Specifically, with the exception of CZL, Asian accessions had significantly (*p* < 0.05) lower average DTF (53.8 days), DTM (113.4 days), and HTFC (90.8 cm) than African accessions (Table 2). Similarly, mean comparisons revealed significantly (*p* < 0.05) higher CZL (106.5 cm), CL (3.1 cm), and TSW (3.4 g) in brown seeds compared to the other seed colors (Table 2). Brown seeds, in addition, recorded the lowest DTF (52.8 days) compared to olive-colored (66.5 days) and white (62.0 days) seeds (*p* < 0.05). Generally, most of the quantitative traits in this study were associated with the continental origin of the accessions. Previously, Frary et al. [50] observed similar findings, while Teboul et al. [53] found the opposite trend. Stavridou et al. [25] further reported dissimilarities in morpho-physiological traits of sesame from different geographical origins. With shorter HTFC and DTM and longer CZL, Asian accessions could produce more capsules per plant and mature earlier than African accessions. Crop species adapt differently to varying environmental conditions, and changes in these conditions can potentially elicit variations in morphological, biochemical, and physiological features of plants [36,37]. Since the location of the experiment was in Asia, the African accessions could have been affected by the difference in environmental conditions. This explains the variation in the quantitative agronomic traits, which seemed to favor the Asian accessions in terms of the number of capsules and maturity. Sa et al. [54] and Desta et al. [55], respectively, observed significant variations in morphological traits of perilla and mung bean accessions from different origins.

The interaction effects of continent and seed color were also statistically analyzed (Table 3 and Table S2). Accordingly, continent and seed color interaction had a significant effect only on DTM and CW, with the other parameters remaining unaffected. A mean comparison of the interaction effect revealed that white seeds from Africa recorded significantly higher DTM (131.7 days) than those from Asia (DTM: 110.8 days) (Table S2). Also, the ANOVA revealed an insignificant influence of the main effect of continent and seed color on CW, but the interaction analysis showed a significant difference between brown (0.9 cm) and white (0.7 cm) seeds from Africa and between white seeds from Africa and Asia (0.8 cm). These findings confirm the significant association of seed color with diverse morphological and physiological plant activities. Even though statistical analysis was not performed, Arteaga et al. [56] observed variation in agronomic traits with respect to black, brown, and white-colored *Phaseolus vulgaris* L. seeds. Brown-colored seeds were characterized by larger seeds compared to the other colored seeds. Yildiz et al. [35] evaluated seed yield parameters of common vetch with different seed colors and reported significant variation in the parameters according to the seed coat colors. Similarly, Ochuodho and Modi [34] observed dormancy in dark-colored mustard seeds against light-colored ones, indicating the association of seed color with physiological activities.

### 3.3. Effects of Origin and Seed Color on Biochemical Parameters

The origin of accessions for different crop species has been reported to be significantly associated with variations in the biochemical properties of their seeds. For sesame seeds, there is limited information on the effect of origin on variations in TPC and antioxidant activities. This study compared variations in TPC and antioxidant activities of sesame seeds from Africa and Asia. Results demonstrated insignificant variability in TPC, ABTS, and DPPH within the sesame germplasm with respect to their continent of origin even though African accessions recorded higher average TPC (31.67 µg GAE/mg DE), ABTS (58.8 µg TE/mg DE), and DPPH (15.5 µg AAE/mg DE) compared to those from Asia (TPC: 25.7 µg GAE/mg DE, ABTS: 48.9 µg TE/mg DE, and DPPH: 13.4 µg AAE/mg DE) (Table 2 and Table 3). The insignificant effect implies that the continent of origin may not serve as a reliable factor for discriminating sesame germplasms based on their biochemical properties.

The interaction effect of continent and seed color was insignificant; nonetheless, the main effect of seed color significantly (*p* < 0.05) influenced diversity in TPC, ABTS, and DPPH activities of the sesame germplasms (Table 2 and Table 3). This observation supports the concept that seed color is among the principal agronomic traits associated with seed biochemical composition [30]. As shown in Table 2, the average TPC decreased in the order of olive-colored (39.7 µg GAE/mg DE) > white (33.3 µg GAE/mg DE) > black (32.0 µg GAE/mg DE) > light brown (19.5 µg GAE/mg DE) > brown (16.1 µg GAE/mg DE) seeds, the former three being significantly different from the latter two seed colors (*p* < 0.05). In a previous study, yellow sorghum seeds recorded significantly higher TPC compared to other seed colors, confirming the association of metabolite composition with seed pigmentation [57]. The average antioxidant activities, including DPPH and ABTS activities, were also highest in olive-colored seeds and lowest in brown seeds. Contrary to Dossou et al. [38] who recorded higher antioxidant activity (DPPH) in black and brown seeds, brown seeds in this study rather recorded the lowest average DPPH scavenging activity (7.9 µg AAE/mg DE) and were statistically different from olive (18.8 µg AAE/mg DE), black (16.5 µg AAE/mg DE), and white (16.2 µg AAE/mg DE) sesame seeds (Table 2 and 3). ABTS scavenging activity also varied among the five seed coat colors in the order of olive (78.9 µg TE/mg DE) > white (64.2 µg TE/mg DE) > black (60.2 µg TE/mg DE) > light brown (37.0 µg TE/mg DE) > brown (25.9 µg TE/mg DE) seeds. Black, white, and olive-colored seeds were all statistically different from brown seeds, but with light brown seeds, statistical difference was only observed with white and olive-colored seeds (Table 2 and Table 3). Previous researchers also compared the antioxidant activity and TPC of different sesame seed coat colors and concluded that the antioxidant activity and TPC of sesame seeds were associated with the color of the seed coat [30,58,59].

### 3.4. Principal Component (PC), Hierarchical Clustering on Principal Component (HCPC), and Correlation Analyses

**Figure 2:**
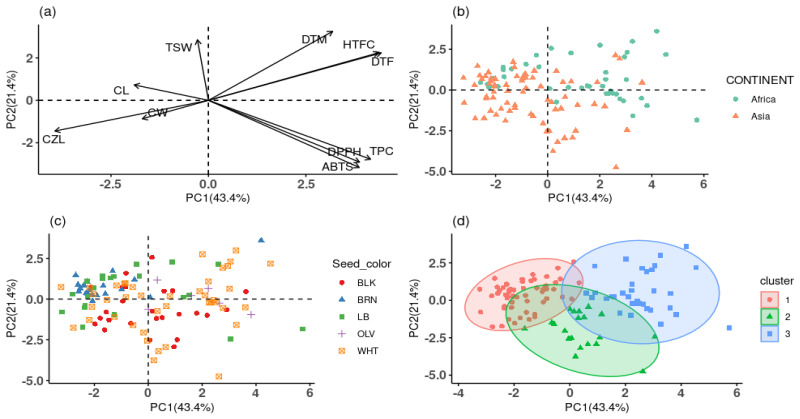
Variable PCA biplot (**a**), score plot of sesame accessions according to continent (**b**) and seed colors (**c**), and hierarchical clustering principal component (HCPC) biplot based on quantitative traits, TPC, ABTS, and DPPH (**d**).

Principal component analysis was performed to ascertain the contribution of each trait to the observed variation in the population. In all, three PCs were identified with eigenvalues ≥1 explaining 75.62% of the overall variation (Table S3). Specifically, the first principal component (PC1) accounted for the highest observed variation of 43.42% with traits such as DTF (16.62%), HTFC (16.13%), ABTS (12.65%), DPPH (12.70%), TPC (14.62%), and CZL (13.13%) contributing highly to the variation. In PC2, which explained 21.43% of the total variation, DTM (18.12%), TSW (13.99%), TPC (13.19%), ABTS (17.16%), and DPPH (14.53%) appeared as the most discriminant traits (Table S3). Overall, traits with higher contributions to PC1 and PC2 are regarded in this study as prominent traits contributing to the variation observed among the accessions and hence could be considered for selection and breeding purposes (Figure 2a and Table S3). In addition, score plots of PC1 and 2 portrayed a rough distribution of the accessions according to the continent of origin (Figure 2b) and seed color (Figure 2c). This confirms the pattern of clustering in Figure 1 and emphasizes the observation that continent and seed color on their own did not introduce much diversity in the natural population of the germplasms.

The biplot of hierarchical clustering on principal components (HCPC) computed with the quantitative agronomic traits, TPC, and antioxidant activities is illustrated in Figure 2d. Overall, the 112 sesame accessions were clustered into three distinct groups with 58, 20, and 34 accessions in clusters 1, 2, and 3, respectively (Figure 2d, Table S4). The ANOVA revealed a significant difference in averages when comparing the variables per cluster. Cluster 1 recorded the highest average of CZL (106.7 cm), CL (2.91 cm), CW (0.85 cm), and TSW (3.16 g), and the difference was significant (*p* < 0.001). Cluster 3 recorded the highest means of DTF (72.06 days), DTM (132.06 days), and HTFC (130.88 cm), which were all significantly (*p* < 0.001) different compared to the other clusters (Table S4). Cluster 2 recorded the highest average of TPC (43.23 µg GAE/mg DE), ABTS (86.96 µg TE/mg DE), and DPPH (21.34 µg AAE/mg DE), followed by cluster 3. The averages of both antioxidant properties in clusters 2 and 3 were significantly (*p* < 0.001) different from cluster 1. Commercial application of sesame seeds as nutraceuticals for the treatment of cancer, respiratory tract infections, reducing blood pressure, and other chronic metabolic diseases were reviewed by Abbas et al. [17]. Sesame accessions from cluster 2 (IT29971, IT185998, and IT104246) and cluster 3 (IT194356, IT170094, and IT169623), which showed higher TPC and antioxidant activities (Table S1), are highly recommended for commercial utilization as nutraceuticals. Their effectiveness can further be evaluated using in vivo biological models.

**Figure 3:**
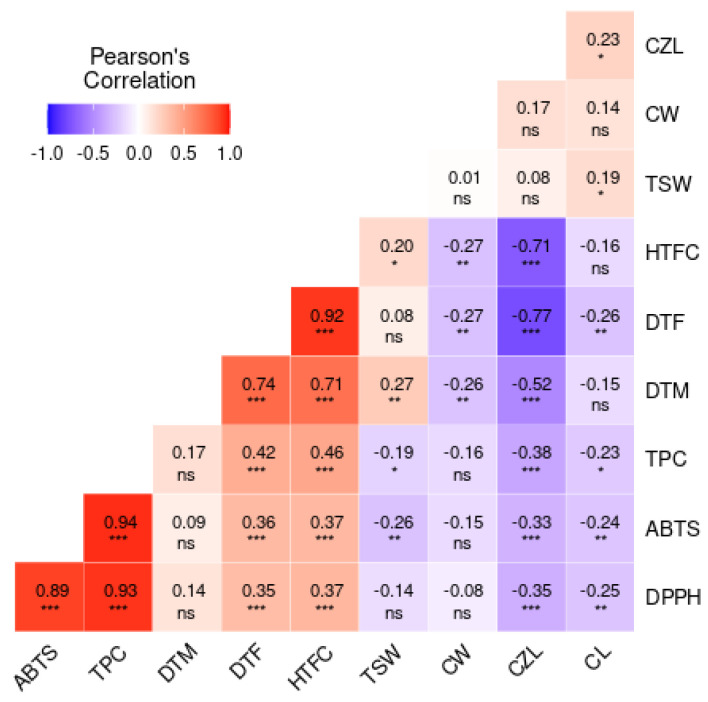
Correlation matrix showing the relationship among quantitative agronomic traits, TPC, and antioxidant activities of sesame; ***, **, *: significant at 0.001, 0.01, and 0.05, respectively, ns: not significant.

For a better understanding of the interrelationship among and between the agronomic and antioxidant traits, a correlation analysis was performed. Results revealed a varying range of correlations between the parameters analyzed (Figure 3). Between TPC and the antioxidant activities, the correlation was significantly (*p* < 0.001) strong and positive (r = 0.89, 0.94, and 0.93, *p* < 0.001, respectively, for ABTS–DPPH, ABTS–TPC, and DPPH–TPC). This explains the reason why accessions with higher TPC recorded higher antioxidant activities (Table S1). Dossou et al. [38] analyzed the correlation between sesame seed antioxidants and phytochemicals. Their findings revealed a significantly high positive correlation between TPC and antioxidant activity, which corroborates the findings of this research. The correlation further supports the PCA biplot in Figure 2a and the heatmap in Figure 1 in which TPC, ABTS, and DPPH traits were clustered together. The positive and/or negative correlations observed among most pairs of the agronomic traits were also significant (*p* < 0.001). DTM positively correlated with DTF (r = 0.74, *p* < 0.001), HTFC (r = 0.71, *p* < 0.001), and TSW (r = 0.27, *p* < 0.01) and negatively correlated with CZL (r = −0.52, *p* < 0.001) and CW (r = −0.26, *p* < 0.01). There was a negative correlation between HTFC and CZL (r = −0.71, *p* < 0.001) (Figure 3). In previous studies analyzing the relationship existing among sesame agronomic traits, similar and contrasting correlations were observed [25,26,28,52,53]. Considering the relationship between agronomic traits, TPC, and antioxidant activities, TSW showed a significant negative correlation with ABTS (r = −0.26, *p* < 0.001,) and TPC (r = −0.19, *p* < 0.05). This correlation suggests that accessions with larger seeds tend to have lower antioxidant activity and reduced TPC. This may explain why brown-colored seeds exhibited the lowest antioxidant activities and TPC, as they also recorded the highest and statistically significant TSW values (Table 2). Seed weight is one of the few agronomic parameters found to influence metabolite composition and antioxidant activity in seeds. Lee et al. [57] evaluated the effect of seed weight on the biochemical content and antioxidant activity of sorghum seeds. Their study found that larger sorghum seeds recorded higher TPC and antioxidant activities, which contrasts with the findings of our current study.

## 4. Conclusions

This research revealed wide inherent diversity in the sesame germplasms with respect to their agronomic traits, polyphenols, and antioxidant activities. Analysis of continental effect and seed color on diversity in sesame polyphenols and antioxidant activities revealed that these properties are independent of geographical location but not seed color. Olive and brown sesame seeds recorded higher and lower means of the biochemical parameters, respectively. Individually, seed color and continent of origin influenced diversity in most of the quantitative agronomic traits, whereas their interaction influenced a few. Specifically, Asian accessions flowered and matured earlier than African accessions, whereas brown and black seeds, respectively, flowered and matured earlier than other seed colors. Correlation analysis revealed a significant association between most pairs of traits including the potential influence of seed weight on polyphenol content and antioxidant activities of sesame seeds. Traits with significant correlations can be utilized as reliable markers for selecting and enhancing traits of interest in sesame breeding programs. Consequently, the sesame population evaluated in this study remains an invaluable resource for direct selection of accessions with high antioxidant activities, which can further be used as donor parents for crosses in breeding high-antioxidant sesame varieties. Overall, this study recommends specific accessions including IT194356, IT170094, IT29971, IT185998, IT104246, and IT169623 for utilization as nutraceutical or functional foods due to their high antioxidant activities. The observed diversity highlights opportunities for future research, particularly in estimating the heritability of traits and conducting genomic studies to explore the underlying genetic mechanisms. Although our study confirms antioxidant activities in vitro through ABTS and DPPH assays, in vivo studies are recommended to further validate these antioxidant potentials under physiological conditions. This would provide a more comprehensive understanding of the accessions’ antioxidant properties.

## Figures and Tables

**Table 1:** Frequency distribution of qualitative agronomic traits within the 112 sesame germplasms from Africa and Asia.

Parameter	Category	Frequency (*f*)		Total (*f*)	Rel. *f* (%)
		Africa	Asia		
Seed color	White	17	24	41	36.6
	Black	4	21	25	22.3
	Olive	4	2	6	5.4
	Brown	6	13	19	16.9
	Light brown	10	11	21	18.8
No. of capsules per axil (NCPA)	1	34	38	72	64.3
	1/3	7	33	40	35.7
No. of locules per capsule (NLPC)	4	40	60	100	89.3
	4/6	0	4	4	3.6
	4/6/8	0	1	1	0.9
	8	0	1	1	0.9
	8/6	1	5	6	5.3

**Table 2:** Diversity and individual effect of continent and seed color on TPC, antioxidant activities, and quantitative agronomic traits of sesame.

Parameter	Value	Continent		Seed Color					Total
		Africa	Asia	Wht	Blk	Olv	Lb	Brn	
DTF (days)	Min.	47.0	44.0	44.0	44.0	54.0	44.0	44.0	44.0
	Max.	97.0	79.0	93.0	70.0	75.0	79.0	97.0	97.0
	Mean	66.5 ^A^	53.8 ^A^	62.0 ^a^	55.9 ^b^	66.5 ^a^	57.3 ^ab^	52.8 ^b^	58.4
	SD	11.9	7.7	12.1	7.5	7.1	10.8	12.0	11.3
	CV								19.4
DTM (days)	Min.	106.0	90.0	90.0	97.0	121.0	93.0	110.0	90.0
	Max.	147.0	147.0	146.0	146.0	138.0	147.0	147.0	147.0
	Mean	126.7 ^A^	113.4 ^B^	119.4 ^a^	114.4 ^ab^	128.8 ^a^	119.2 ^a^	116.4 ^ab^	118.3
	SD	11.1	12.0	15.7	11.7	7.3	13.8	8.2	13.3
	CV								11.4
HTFC (cm)	Min.	27.6	12.2	15.7	23.5	51.0	12.2	27.6	12.2
	Max.	215.0	153.2	215.0	137.0	144.8	152.3	211.8	215.0
	Mean	114.1 ^A^	57.9 ^B^	90.8 ^a^	70.2 ^ab^	105.2 ^a^	68.9 ^ab^	65.0 ^ab^	78.5
	SD	46.1	29.3	52.8	30.6	35.5	44.8	40.3	45.2
	CV								57.5
CZL (cm)	Min.	25.0	33.4	25.0	42.6	37.4	35.7	28.1	25.0
	Max.	144.2	151.4	147.8	148.4	111.5	151.4	129.0	151.4
	Mean	71.1 ^B^	98.8 ^A^	78.3 ^b^	92.7 ^ab^	70.2 ^b^	93.1 ^ab^	106.5 ^a^	88.7
	SD	30.2	29.3	35.1	28.4	25.4	31.6	25.0	32.4
	CV								36.7
CL (cm)	Min.	2.4	2.1	2.1	2.2	2.3	2.2	2.5	2.1
	Max.	3.3	4.1	4.1	3.3	3.0	3.3	3.6	4.1
	Mean	2.7 ^A^	2.8 ^A^	2.8 ^b^	2.6 ^b^	2.7 ^b^	2.7 ^b^	3.0 ^a^	2.8
	SD	0.2	0.3	0.3	0.3	0.2	0.2	0.2	0.3
	CV								12.2
CW (cm)	Min.	0.5	0.5	0.5	0.7	0.7	0.5	0.5	0.5
	Max.	1.0	1.2	1.1	1.1	0.8	1.2	1.0	1.2
	Mean	0.7 ^A^	0.8 ^A^	0.7 ^a^	0.7 ^a^	0.7 ^a^	0.8 ^a^	0.8 ^a^	0.8
	SD	0.1	0.1	0.1	0.1	0.0	0.1	0.1	0.1
	CV								17.0
TSW (g)	Min.	2.3	1.8	2.1	2.4	2.6	1.8	2.8	1.8
	Max.	4.4	3.8	3.8	4.0	3.4	4.2	4.4	4.4
	Mean	3.1 ^A^	2.9 ^A^	2.9 ^b^	3.0 ^b^	3.0 ^ab^	3.0 ^b^	3.3 ^a^	3.0
	SD	0.5	0.4	0.3	0.4	0.3	0.5	0.3	0.4
	CV								14.8
TPC (µg GAE/mg DE)	Min.	5.1	5.0	5.0	8.3	23.6	5.1	7.3	4.9
	Max.	87.9	69.3	69.3	52.3	61.8	87.9	38.9	87.9
	Mean	31.6 ^A^	25.6 ^A^	33.3 ^a^	32.0 ^a^	39.7 ^a^	19.5 ^b^	16.1 ^b^	27.9
	SD	17.7	15.2	14.0	14.2	13.4	20.4	8.0	16.4
	CV								58.9
ABTS (µg TE/mg DE)	Min.	8.6	3.4	11.1	9.4	50.6	8.6	3.4	3.4
	Max.	176.0	175.0	175.0	111.5	108.9	176.0	60.8	176.0
	Mean	58.8 ^A^	48.9 ^A^	64.2 ^a^	60.2 ^a^	78.9 ^a^	37.0 ^b^	25.9 ^b^	52.6
	SD	37.9	33.0	33.6	31.7	23.7	38.8	15.6	35.1
	CV								66.5
DPPH (µg AAE/mg DE)	Min.	4.0	4.0	4.7	4.6	11.0	4.0	4.0	3.9
	Max.	46.2	37.3	37.3	29.1	26.3	46.2	18.2	46.2
	Mean	15.5 ^A^	13.3 ^A^	16.2 ^a^	16.5 ^a^	18.8 ^a^	11.3 ^b^	7.9 ^b^	14.1
	SD	8.6	7.5	6.9	7.4	5.8	10.1	4.0	8.0
	CV								56.8

Different superscript letters shows significant difference in means per factor for each parameter, DTF: days to flowering, DTM: days to maturity, HTFC: height to first capsule, CZL: capsule zone length, CL: capsule length, CW: capsule width, TSW: thousand seed weight, TPC: total phenolic content, DPPH: 2,2-diphenyl-1-picrylhydrazyl, ABTS: 2,2′-azinobis-(3-ethylbenzo thiazoline-6-sulfonic acid), SD: standard deviation, CV: coefficient of variation, Blk: black, Brn: brown, Lb: light brown, Olv: olive, Wht: white.

**Table 3:** Analysis of variance for the 112 sesame germplasms based on continental origin and seed color.

Factor	TPC	ABTS	DPPH	DTF	DTM	HTFC	CZL	CL	CW	TSW
Continent	ns	ns	ns	***	***	***	***	ns	ns	ns
Seed color	***	***	***	*	ns	ns	*	**	ns	**
Continent–seed color	ns	ns	ns	ns	*	ns	ns	ns	*	ns

TPC: total polyphenol content, ABTS: 2,2′-azinobis-(3-ethylbenzo thiazoline-6-sulfonic acid), DPPH: 2,2-diphenyl-1-picrylhydrazyl, DTF: days to flowering, DTM: days to maturity, HTFC: height to first capsule, CZL: capsule zone length, CL: capsule length, CW: capsule width, TSW: thousand seed weight, ***, **, *: significant at 0.001, 0.01, and 0.05, respectively, ns: not significant.

## Data Availability

The original contributions presented in the study are included in the article/Supplementary Materials, further inquiries can be directed to the corresponding author.

## Supplementary Materials

The following supporting information can be downloaded at: https://www.mdpi.com/article/10.3390/foods13182932/s1, Table S1: Sesame accessions used in this study and their contribution to the quantitative agronomic traits and antioxidant properties in the clusters; Table S2: Interaction effect of continent and seed color on TPC, antioxidant activities, and quantitative agronomic traits of sesame; Table S3: Contribution and correlation of traits to the first five principal components (PCs); Table S4: Number of accessions in clusters and the contribution of quantitative and antioxidant traits to each cluster.
