# Supplementary material for: Evaluation of Agronomic Traits, Total Phenolic Content, and Antioxidant Properties of Sesame Seeds of Different Colors and Origin
Source: Foods. 2024 Sep 16;13(18):2932. doi: 10.3390/foods13182932 (PMC11431559; doi:10.3390/foods13182932)
Supplement: Supplementary file 1 [file foods-13-02932-s001.zip › foods-3191609-supplementary.pdf]

Table S1. Sesame accessions used in the study and their contribution to the quantitative agronomic traits and antioxidant properties in the clusters

Accession	Continent	Seed color	Clusters	DTF (day)	DTM (day)	HTFC (cm)	CZL (cm)	CL (cm)	CW (cm)	TSW (g)	ABTS (μg TE/ mg DE)	DPPH (μg AAE/ mg DE)	TPC (μg GAE/ mg DE)
IT218263	Africa	Blk	1	49	113	49.6	110	2.8	0.9	3.7	19.1	8.8	10.7
IT184553	Africa	Blk	1	65	113	137	53.2	2.5	0.7	4	9.4	7.5	12.1
IT184738	Africa	Blk	1	65	125	92	71.3	2.9	1	2.8	11.3	4.6	10
IT170011	Africa	Blk	3	65	126	107	56.6	2.6	0.7	3.4	107.4	26.7	51.4
IT167146	Africa	Brn	3	97	147	211.9	28.1	2.8	0.9	4.4	60.8	18.2	38.9
IT311597	Africa	Brn	1	54	110	79	111.4	2.9	0.8	3.2	11.2	5	17.7
IT238548	Africa	Brn	1	47	110	50.5	115	3.3	1	3.5	23.2	5.6	17.1
IT238549	Africa	Brn	1	49	110	69.2	123.7	3.4	1	3.3	36	5.7	14.3
IT242948	Africa	Brn	1	49	113	27.6	63	2.7	1	2.8	13.3	8	7.3
IT184554	Africa	Brn	1	57	113	89.2	89	2.8	0.8	3.4	55.1	14.6	30.3
IT194356	Africa	Lb	3	75	147	152.3	115.6	2.4	0.9	4.2	176	46.2	87.9
IT169615	Africa	Lb	1	54	124	45.3	91.5	2.6	0.9	3.1	12.6	4	5.1
IT271182	Africa	Lb	1	54	106	74.3	77	3	0.9	3.9	8.6	7.3	12.8
IT219069	Africa	Lb	1	47	113	29.5	92	2.8	1	3.1	27.8	6.5	9.4
IT271209	Africa	Lb	1	54	120	57	108	2.6	0.7	3.5	11.9	7.6	10.6
IT170078	Africa	Lb	1	61	125	99.2	88	2.8	0.7	2.9	23.6	5.8	15.8
IT170100	Africa	Lb	3	65	125	106.8	83.2	2.7	0.7	2.8	56.4	16.6	36.8
IT170102	Africa	Lb	3	70	125	102.6	58.8	2.5	0.7	2.5	51.9	15.3	30.3
IT170135	Africa	Lb	1	65	138	125.8	123.7	3.3	0.9	3.4	32.7	5.3	19.3
IT170145	Africa	Lb	3	75	126	122.3	35.8	2.8	0.6	2.6	22.8	6	7.5
IT169623	Africa	Olv	3	75	126	144.8	37.4	2.9	0.8	2.6	108.9	26.3	61.8
IT170104	Africa	Olv	3	65	138	105.8	73	2.7	0.7	3.2	99.8	24.3	47.9
IT170114	Africa	Olv	3	65	125	119.3	59.8	2.8	0.7	2.8	62.8	19	33
IT170123	Africa	Olv	3	70	138	133.7	83.1	2.8	0.8	3.4	89.9	18.5	39.5
IT184342	Africa	Wht	3	82	146	179	33	2.7	0.6	3.5	42.4	15	31.5
IT184344	Africa	Wht	3	82	127	195	28	2.8	0.8	3.3	40.4	17.4	35.5
IT184345	Africa	Wht	3	93	139	215	25	2.5	1	3.8	75.6	21.9	45.2
IT184346	Africa	Wht	3	82	139	198	38	3	0.8	3.2	56.8	15.5	32.7
IT167148	Africa	Wht	3	61	126	85.2	96.6	2.7	0.8	3.7	66.1	19.3	38.2
IT169622	Africa	Wht	3	79	146	174	49	3.1	0.9	3.2	43.2	10.1	24.2
IT169726	Africa	Wht	2	54	125	84.4	144.2	2.5	0.6	3	60.7	19	39.1
IT170077	Africa	Wht	3	75	125	110.2	56.2	3.1	0.7	2.8	87.4	21.3	46.2
IT170081	Africa	Wht	3	75	138	122.2	46.8	2.6	0.7	2.5	71.7	12	27.3
IT170082	Africa	Wht	3	75	125	114.8	55.6	2.6	0.7	2.4	93.8	20	43.8
IT170083	Africa	Wht	3	70	127	112.8	60.4	2.6	0.6	2.4	75.8	19	39.1
IT170094	Africa	Wht	3	65	125	119.6	65.6	2.7	0.7	2.8	125.6	26.8	61.4
IT170107	Africa	Wht	3	75	138	115.6	46.4	2.5	0.7	3	79.5	21.6	38.3
IT170110	Africa	Wht	3	75	138	121.6	62.8	2.6	0.7	2.6	105.1	26.4	49.4
IT170115	Africa	Wht	3	65	125	144.4	50.2	2.8	0.7	2.7	91.3	17.9	40.2
IT170150	Africa	Wht	3	65	125	123.4	55	2.7	0.6	2.5	80.2	19	38.5
IT170030	Africa	Wht	3	65	125	133.4	57.6	2.9	0.7	3.2	83.9	20.1	40.2
IT286174	Asia	Blk	2	44	100	42.8	148.4	3.4	0.8	3.2	69.5	18.8	37.3

IT312163	Asia	Blk	2	54	119	77.6	100.3	3	0.9	2.7	71.9	18.5	41.3
IT275748	Asia	Blk	2	44	106	61.4	108	3.2	0.8	3.1	72.2	17.8	40.4
IT314517	Asia	Blk	1	49	110	51.5	134.5	2.9	0.7	2.7	39	11.2	25.6
IT277623	Asia	Blk	1	54	100	33	128.5	2.7	0.9	3	67.3	10.8	24.8
IT169365	Asia	Blk	2	54	121	50.5	62	2.4	0.9	2.5	63.8	17.7	33.6
IT169131	Asia	Blk	3	61	124	73	86.3	2.3	0.7	3.5	45.8	15.4	31
IT169441	Asia	Blk	3	70	146	126.8	42.6	2.2	0.8	3.3	66.2	29.1	49.3
IT169442	Asia	Blk	3	54	126	76	82.6	2.2	0.7	3.7	93.2	25.7	46.4
IT283740	Asia	Blk	2	54	113	61.3	72.6	2.7	0.7	2.8	69.7	13.5	32.8
IT297185	Asia	Blk	1	54	110	57.7	113	2.5	0.7	3.2	25.8	18.2	13.8
IT267681	Asia	Blk	1	57	113	78.5	88.5	2.9	0.7	3	21.8	8.8	16.1
IT105793	Asia	Blk	2	54	121	73	77.8	2.4	0.7	3.1	81.6	21.8	36.9
IT113593	Asia	Blk	2	54	97	76.8	83.3	2.6	0.9	3.2	107.4	26.6	49.3
IT103367	Asia	Blk	2	54	110	23.5	112.5	3.1	0.8	2.5	100.3	22	43.2
IT104246	Asia	Blk	2	54	106	49.6	89.8	2.9	0.8	2.5	111.5	26.5	49.4
IT221528	Asia	Blk	1	47	100	28.2	118.4	2.5	0.8	2.8	34.3	10.1	25.3
IT221525	Asia	Blk	1	47	97	27.2	140.2	2.3	0.8	2.8	36.1	9.8	28.4
IT184696	Asia	Blk	3	70	125	113	66.6	2.9	1.1	2.9	97	24	52.3
IT170033	Asia	Blk	1	61	113	95.2	101	2.6	0.7	2.7	34.5	4.6	8.3
IT194014	Asia	Blk	3	65	127	94.3	69.6	3	0.8	2.9	49	16.4	30.3
IT242902	Asia	Brn	1	47	120	57.5	110.5	3.4	1	3.7	3.4	5.7	8.7
IT311685	Asia	Brn	1	47	113	41.6	121.3	2.8	0.8	3.1	20	4.7	13.1
IT184505	Asia	Brn	1	61	120	86.6	98.8	3.6	0.8	3.4	17.3	8.7	9.8
IT217022	Asia	Brn	1	49	119	52.3	101.6	3.4	0.8	3.1	7.8	4	12.9
IT311689	Asia	Brn	1	47	119	35	129	3.2	0.8	3.3	11.7	4.6	10.3
IT184290	Asia	Brn	1	65	113	82	113.7	2.5	0.6	3.5	45.1	5.4	13.8
IT271249	Asia	Brn	1	54	113	77.4	110.2	2.9	0.8	3.6	25.9	12.8	23.8
IT242909	Asia	Brn	1	47	113	58.8	94.2	3.3	1	3.2	22	6.2	14.3
IT169236	Asia	Brn	1	47	121	45.5	124.8	2.9	0.8	3.5	24.4	11.3	23.5
IT169406	Asia	Brn	1	47	113	31.8	128.5	2.9	0.8	3.8	25.4	7.2	9.1
IT271222	Asia	Brn	1	44	113	40	128.5	3.2	0.8	3.3	29.9	13.1	17.4
IT271236	Asia	Brn	1	47	120	41.6	119.8	3.1	0.8	3.2	18.8	4.2	9.5
IT242911	Asia	Brn	1	49	113	58.2	112.8	3.2	1	3.3	41.7	6.8	14.8
IT300111	Asia	Lb	1	47	113	32.3	142.3	3.3	0.8	3.3	15.1	8.2	12.7
IT195853	Asia	Lb	3	79	147	141	38	2.3	0.7	2.6	53.3	10	20.3
IT28914	Asia	Lb	1	44	113	15.4	136.4	2.6	1.3	2.6	18.9	23.9	9.5
IT169578	Asia	Lb	1	47	97	12.2	97.6	2.2	0.7	2.8	22.6	4.6	6.9
IT169800	Asia	Lb	1	49	121	23.8	151.4	2.7	0.9	2.6	30.7	5.2	15
IT184530	Asia	Lb	1	57	125	44.4	81.8	2.8	0.7	3	9.5	5.8	7.8
IT29100	Asia	Lb	1	47	93	22.3	91.5	2.8	1.2	2.7	18	5.4	5.2
IT29940	Asia	Lb	1	54	113	47.6	90.4	2.3	1.2	2.5	41.4	10.8	17.9
IT170031	Asia	Lb	2	65	113	111.6	46.4	2.6	0.7	1.8	105.3	25.9	62
IT169643	Asia	Lb	1	49	110	31.6	95	2.9	0.8	3.9	22.1	4.5	8
IT271228	Asia	Lb	1	47	110	50.8	112.8	3.1	0.8	3.8	17.6	14.3	9
IT169801	Asia	Olv	1	70	121	77	56.8	3	0.9	3.5	50.6	11	23.6
IT186006	Asia	Olv	2	54	125	51	111.5	2.3	0.8	2.9	61.6	13.8	32.7
IT220658	Asia	Wht	1	44	92	15.8	131.5	3.1	1	2.9	45.7	10.2	25.7
IT275747	Asia	Wht	3	75	138	153.2	42	2.4	0.7	3.6	66.9	13.4	34.9
IT169337	Asia	Wht	1	57	125	66	80.6	4.2	0.7	3.1	35.2	12.7	23.6
IT184445	Asia	Wht	3	70	120	102	35	2.4	0.7	3	60.2	14.2	37.7

IT242903	Asia	Wht	1	47	113	24.6	119.8	3.6	0.9	3.3	11.1	5.7	5
IT312164	Asia	Wht	1	49	106	52.8	132	3	0.9	3.1	25.2	11.9	21.3
IT170202	Asia	Wht	1	54	125	18.8	33.4	3.2	0.9	3.3	21.3	15.1	22.9
IT184531	Asia	Wht	1	57	113	49	97.4	2.6	1	2.6	16.3	7.3	17.5
IT184524	Asia	Wht	2	57	120	94	58.5	2.6	1	2.8	51.8	17.6	34.9
IT192444	Asia	Wht	1	57	125	73.7	101.5	3.5	0.7	3.2	47.2	9	19
IT267641	Asia	Wht	2	57	97	66.3	116.6	3.1	0.8	2.7	59.6	18.4	36.2
IT192437	Asia	Wht	1	54	125	68.2	103.6	3.1	0.9	2.9	46.4	10.9	24.7
IT29971	Asia	Wht	2	54	93	62.2	60.8	2.8	0.9	2.6	175	37.3	69.3
IT103159	Asia	Wht	2	49	90	56	73	3.1	0.8	2.3	107.8	24.2	45.6
IT103957	Asia	Wht	2	54	90	34.5	93.8	2.9	0.9	2.2	105.1	25.1	50.4
IT104927	Asia	Wht	1	57	121	72	79.3	2.7	0.8	2.8	42.4	10.6	20.9
IT185998	Asia	Wht	2	49	92	77.2	92.8	3.2	0.8	3	112.5	25.9	56.2
IT160628	Asia	Wht	2	54	110	52.3	91	2.2	1.1	2.3	69.1	16.6	32.4
IT28892	Asia	Wht	2	54	100	50.8	82.2	2.6	1.1	2.5	82.7	19.8	41.5
IT189651	Asia	Wht	1	54	113	52.5	132.5	2.7	0.8	2.9	30.4	4.8	7.7
IT192267	Asia	Wht	1	47	125	19.5	114	2.9	0.8	3.1	22	4.7	10.1
IT221527	Asia	Wht	1	49	100	23.2	147.8	2.5	0.7	2.6	44.7	7.8	18.2
IT326979	Asia	Wht	1	49	106	44.5	135.2	2.5	0.8	3.1	61.4	11.9	26.9
IT166731	Asia	Wht	1	57	121	46	89.5	2.7	0.8	3	16.5	7.8	13.6

DTF: Days to Flowering, DTM: Days to Maturity, HTFC: Height to First Capsule, CZL: Capsule Zone Length, CL: Capsule Length, CW: Capsule width, TSW: Thousand Seed Weight, TPC: Total Polyphenol Content, ABTS: 2,2'-azinobis-(3-ethylbenzo thiazoline-6-sulfonic acid), DPPH: 2,2-diphenyl-1-picrylhydrazyl, Blk: black, Brn: brown, Lb: light brown, Olv: olive, Wht: white.

Table S2. Interaction effect of continent and seed color on TPC, antioxidant activities, and quantitative agronomic traits of sesame

Parameter	Continent	Seed color	*n*	Min.	Max.	Mean±SD
TPC (μg GAE/mg DE)	Africa	White	17	24.2	61.4	39.5±8.6 [a]
		Black	4	10	51.4	23.6±21.1 [bcd]
		Olive	4	33.0	61.8	45.6±12.4 [a]
		Brown	6	7.3	38.9	20.9±11.5 [cd]
		Light brown	10	5.1	87.9	23.6±24.8 [bcd]
	Asia	White	24	5.0	69.3	29.0±15.6 [bc]
		Black	21	8.3	52.3	34.1±12.4 [ab]
		Olive	2	23.6	32.7	28.2±6.4 [bcd]
		Brown	13	8.7	23.8	13.9±5.0 [d]
		Light brown	11	5.2	62.0	15.9±16.0 [d]
ABTS (μg TE/ mg DE)	Africa	White	17	40.4	125.6	75.23±22.7 [ab]
		Black	4	9.4	107.4	36.8±47.3 [cde]
		Olive	4	62.8	108.9	90.4±19.9 [a]
		Brown	6	11.2	60.8	33.3±21.1 [de]
		Light brown	10	8.6	176.0	42.4±49.6 [cde]
	Asia	White	24	11.1	175.0	56.5±38.3 [bcd]

		Black	21	21.8	111.5	64.7±27.3 ^{abc}
		Olive	2	50.6	61.6	56.1±7.8 ^{bcde}
		Brown	13	3.4	45.1	22.6±11.9 ^{e}
		Light brown	11	9.5	105.3	32.2±27.3 ^{e}
DPPH (µg AAE/ mg DE)	Africa	White	17	10.1	26.8	18.9±4.4 ^{a}
		Black	4	4.6	26.7	11.9±10.0 ^{bcd}
		Olive	4	18.5	26.3	22.0±3.9 ^{a}
		Brown	6	5.0	18.2	9.5±5.5 ^{cd}
		Light brown	10	4.0	46.2	12.1±12.7 ^{bcd}
	Asia	White	24	4.7	37.3	14.3±7.8 ^{bc}
		Black	21	4.6	29.1	17.5±6.8 ^{ab}
		Olive	2	11.0	13.8	12.4±1.9 ^{bcd}
		Brown	13	4.0	13.1	7.3±3.2 ^{d}
		Light brown	11	4.5	25.9	10.8±7.6 ^{cd}
DTF (days)	Africa	White	17	54	93	72.8±9.6 ^{a}
		Black	4	49	65	61.0±8.0 ^{bc}
		Olive	4	65	75	68.8±4.8 ^{ab}
		Brown	6	47	97	58.8±19.1 ^{bc}
		Light brown	10	47	75	62.0 ± 9.6 ^{b}
	Asia	White	24	44	75	54.4 ± 6.9 ^{c}
		Black	21	44	70	55.0 ± 7.2± ^{c}
		Olive	2	54	70	62.0 ± 11.3 ^{bc}
		Brown	13	44	65	50.1 ± 6.2 ^{c}
		Light brown	11	44	79	53.2 ± 10.5 ^{c}
DTM (days)	Africa	White	17	125	146	131.7 ± 8.0 ^{a}
		Black	4	113	126	119.3 ± 7.2 ^{abc}
		Olive	4	125	138	131.8 ± 7.2 ^{a}
		Brown	6	110	147	117.2 ± 14.7 ^{abc}
		Light brown	10	106	147	124.9 ± 11.5 ^{ab}
	Asia	White	24	90	138	110.8 ± 13.9 ^{c}
		Black	21	97	146	113.5 ± 12.3 ^{c}
		Olive	2	121	125	123.0 ± 2.8 ^{abc}
		Brown	13	113	121	116.2 ± 3.6 ^{bc}
		Light brown	11	93	147	114.1 ± 14.2 ^{c}
HTFC (cm)	Africa	White	17	84.4	215.0	138.2 ± 39.5 ^{a}
		Black	4	49.6	137.0	96.4 ± 36.4 ^{bc}

		Olive	4	105.8	144.8	125.9 ± 16.9 [ab]
		Brown	6	27.6	211.8	87.9 ± 64.6 [bcd]
		Light brown	10	29.5	152.3	91.5 ± 39.0 [bc]
		White	24	15.8	153.2	57.3 ± 30.6 [de]
		Black	21	23.5	126.8	65.3 ± 27.7 [cde]
	Asia	Olive	2	51.0	77.0	64.0 ± 18.4 [cde]
		Brown	13	31.7	86.6	54.4 ± 17.9 [de]
		Light brown	11	12.2	141.0	48.4 ± 41.0 [e]
CZL (cm)	Africa	White	17	25.0	144.2	57.0 ± 27.8 [d]
		Black	4	53.2	110.0	72.7 ± 26.0 [bcd]
		Olive	4	37.4	83.1	63.3 ± 19.7 [cd]
		Brown	6	28.1	123.7	88.3 ± 36.7 [bc]
		Light brown	10	35.7	123.7	87.3 ± 26.2 [bc]
	Asia	White	24	33.4	147.8	93.4 ± 32.2 [bc]
		Black	21	42.6	148.4	96.4 ± 27.8 [ab]
		Olive	2	56.8	111.5	84.1 ± 38.6 [bcd]
		Brown	13	94.2	129.0	114.8 ± 11.7 [a]
		Light brown	11	38.0	151.4	98.5 ± 36.2 [ab]
CL (cm)	Africa	White	17	2.4	3.1	2.7 ± 0.1 [b]
		Black	4	2.5	2.9	2.7 ± 0.1 [b]
		Olive	4	2.6	2.8	2.7 ± 0.1 [ab]
		Brown	6	2.6	3.3	2.9 ± 0.3 [ab]
		Light brown	10	2.4	3.3	2.7 ± 0.2 [b]
	Asia	White	24	2.1	4.1	2.8 ± 0.4 [ab]
		Black	21	2.2	3.3	2.6 ± 0.3 [b]
		Olive	2	2.3	3.0	2.6 ± 0.5 [b]
		Brown	13	2.5	3.6	3.0 ± 0.3 [a]
		Light brown	11	2.2	3.2	2.6 ± 0.3 [b]
CW (cm)	Africa	White	17	0.5	0.9	0.7 ± 0.1 [b]
		Black	4	0.7	1.0	0.8 ± 0.1 [ab]
		Olive	4	0.7	0.8	0.7 ± 0.1 [ab]
		Brown	6	0.7	1.0	0.9 ± 0.1 [a]
		Light brown	10	0.5	1.0	0.7±0.1 [ab]
	Asia	White	24	0.6	1.1	0.8 ± 0.1 [a]
		Black	21	0.7	1.1	0.7 ± 0.1 [ab]
		Olive	2	0.7	0.8	0.8 ± 0.1 [ab]

		Brown	13	0.5	1.0	0.8 ± 0.1 ab
		Light brown	11	0.6	1.2	0.8 ± 0.2 a
TSW (g)	Africa	White	17	2.3	3.8	2.9 ± 0.4 bc
		Black	4	2.8	4.0	3.4 ± 0.5 a
		Olive	4	2.6	3.3	2.9 ± 0.3 abc
		Brown	6	2.8	4.4	3.4 ± 0.5 a
		Light brown	10	2.4	4.2	3.1 ± 0.5 ab
	Asia	White	24	2.1	3.6	2.8 ± 0.3 c
		Black	21	2.4	3.6	2.9 ± 0.3 bc
		Olive	2	2.9	3.4	3.1 ± 0.3 abc
		Brown	13	3.1	3.7	3.3 ± 0.2 a
		Light brown	11	1.8	3.8	2.8 ± 0.6 c

TPC: Total Polyphenol Content, ABTS: 2,2'-azinobis-(3-ethylbenzo thiazoline-6-sulfonic acid), DPPH: 2,2-diphenyl-1-picrylhydrazyl, DTF: Days to Flowering, DTM: Days to Maturity, HTFC: Height to First Capsule, CZL: Capsule Zone Length, CL: Capsule Length, CW: Capsule width, TSW: Thousand Seed Weight, SD: Standard Deviation, CV: Coefficient of Variation, different letters in a column indicate significant difference in means for each parameter.

Table S3. Contribution and correlation of traits to the first 5 Principal Components (PC)

Variables	PC1	PC2	PC3	PC4	PC5
%Contribution of traits to PC					
DTF	16.62	8.58	0.24	1.94	0.75
DTM	8.58	18.12	0.02	0.00	0.53
HTFC	16.13	8.64	0.96	0.23	0.52
CZL	13.13	3.59	1.58	10.57	7.18
CL	3.07	0.90	38.32	2.64	53.95
CW	2.44	1.29	16.42	74.19	3.14
TSW	0.07	13.99	31.22	7.99	32.21
ABTS	12.65	17.16	2.01	1.10	0.00
DPPH	12.70	14.53	5.37	0.30	1.67
TPC	14.62	13.19	3.87	1.04	0.05
Correlation between traits and PCs					
DTF	0.85	0.43	-0.05	0.13	-0.08
DTM	0.61	0.62	0.01	0.00	0.06
HTFC	0.84	0.43	0.10	0.05	-0.06
CZL	-0.76	-0.28	0.13	-0.31	0.23
CL	-0.36	0.14	0.64	-0.15	-0.64
CW	-0.33	-0.17	0.42	0.81	0.15
TSW	-0.05	0.55	0.58	-0.27	0.49
ABTS	0.74	-0.61	0.15	-0.10	0.00
DPPH	0.74	-0.56	0.24	-0.05	0.11
TPC	0.80	-0.53	0.20	-0.10	0.02
Eigenvalue	4.30	2.12	1.07	0.88	0.75
Variance (%)	43.42	21.43	10.76	8.87	7.59
Cumulative (%)	43.42	64.85	75.62	84.49	92.08

DTF: Days to Flowering, DTM: Days to Maturity, HTFC: Height to First Capsule, CZL: Capsule Zone Length, CL: Capsule Length, CW: Capsule width, TSW: Thousand Seed Weight, TPC: Total Polyphenol Content, ABTS: 2,2'-azinobis-(3-ethylbenzo thiazoline-6-sulfonic acid), DPPH: 2,2-diphenyl-1-picrylhydrazyl

Table S4. Number of accessions in clusters and the contribution of quantitative and antioxidant traits to each cluster

Clusters	Total Acc.	DTF	DTM	HTFC	CZL	CL	CW	TSW	ABTS	DPPH	TPC
1	58	52.29 ^{b}	114.00 ^{b}	53.25 ^{b}	106.70 ^{a}	2.91 ^{a}	0.85 ^{a}	3.16 ^{a}	26.90 ^{b}	8.42 ^{b}	15.32 ^{b}
2	20	53.35 ^{b}	107.40 ^{c}	62.84 ^{b}	91.27 ^{b}	2.77 ^{ab}	0.84 ^{ab}	3.08 ^{a}	86.96 ^{a}	21.34 ^{a}	43.23 ^{a}
3	34	72.06 ^{a}	132.06 ^{a}	130.88 ^{a}	56.48 ^{c}	2.66 ^{b}	0.75 ^{b}	2.68 ^{b}	76.09 ^{a}	19.66 ^{a}	40.26 ^{a}
ANOVA		***	***	***	***	***	***	***	***	***	***

DTF: Days to Flowering, DTM: Days to Maturity, HTFC: Height to First Capsule, CZL: Capsule Zone Length, CL: Capsule Length, CW: Capsule width, TSW: Thousand Seed Weight, TPC: Total Polyphenol Content, ABTS: 2,2'-azinobis-(3-ethylbenzo thiazoline-6-sulfonic acid), DPPH: 2,2-diphenyl-1-picrylhydrazyl
